# Novel contributors to B cell activation during inflammatory CNS demyelination; An oNGOing process

**DOI:** 10.7150/ijms.66350

**Published:** 2022-01-01

**Authors:** Olympia Damianidou, Paschalis Theotokis, Nikolaos Grigoriadis, Steven Petratos

**Affiliations:** 1 B' Department of Neurology, Laboratory of Experimental Neurology and Neuroimmunology, AHEPA University Hospital, Thessaloniki 54636, Macedonia, Greece.; 2 Department of Neuroscience, Central Clinical School, Monash University, Prahran, Victoria 3004, Australia.

**Keywords:** B cell-activating factor, Nogo receptor 1, autoimmune demyelination

## Abstract

Over the past two decades, the development of targeted immunotherapeutics for relapsing-remitting multiple sclerosis has been successfully orchestrated through the efficacious modulation of neuroinflammatory outcomes demonstrated in the experimental autoimmune encephalomyelitis (EAE) model. In this model, the focus of developing immunomodulatory therapeutics has been demonstrated through their effectiveness in modifying the pro-inflammatory Th1 and Th17-dependent neuropathological outcomes of demyelination, oligodendrocytopathy and axonal dystrophy. However, recent successful preclinical and clinical trials have advocated for the significance of B cell-dependent immunopathogenic responses and has led to the development of novel biologicals that target specific B cell phenotypes. In this context, a new molecule, B-cell activating factor (BAFF), has emerged as a positive regulator of B cell survival and differentiation functioning through various signaling pathways and potentiating the activity of various receptor complexes through pleiotropic means. One possible cognate receptor for BAFF includes the Nogo receptor (NgR) and its homologs, previously established as potent inhibitors of axonal regeneration during central nervous system (CNS) injury and disease. In this review we provide current evidence for BAFF-dependent signaling through the NgR multimeric complex, elucidating their association within the CNS compartment and underlying the importance of these potential pathogenic molecular regulators as possible therapeutic targets to limit relapse rates and potentially MS progression.

## Introduction

Multiple Sclerosis (MS) is a chronic inflammatory and demyelinating disease of the central nervous system (CNS) with hallmarks of myelin degeneration and axonopathy 1. Although its actual cause remains still unknown, various environmental factors such as vitamin D deficiency, smoking, Epstein-Barr virus infection, and genetic factors have been accused as risk factors of the disease 1.

MS is a heterogeneous disease but presentation of characteristic symptoms generally follow stages of neurological deficits (relapses) and these can be reversed during periods of total or partial recovery (remissions) 2, 3. This clinical presentation is seen in relapsing remitting MS (RRMS) that is the most common (85% of individuals diagnosed) form of the disease 1, 2. Gradually, the majority of patients with RRMS will transition into secondary progressive MS (SPMS) having accumulated neural tissue damage and hence disability 2. In some individuals progressive disease manifests in the initial phases of MS without remissions (primary progressive MS; PPMS) resulting in permanent neurodegeneration and in the rare case of progressive relapsing MS (PRMS) the initial progressive phase is followed by acute relapses with intervals of continuous progression 1, 2. Finally, the inflammatory and demyelinating findings in clinically isolated syndrome (CIS) and the evidence in radiologically isolated syndrome (RIS) are supportive of MS in undiagnosed individuals 2, 3.

Numerous clinical trials conducted in participants living with relapsing MS and PPMS have contributed to the understanding of MS pathogenesis allowing for development and management of this heterogeneous disease through targeted disease modifying therapies (DMTs) as the stochastic nature of symptoms evolve 4. Such trials have contributed to the transition from the original dogma that T cell-dependent mechanisms should be the target of therapeutics, into a more dynamic model of complex B and T cell interactions, contributing to the current B cell depleting trials which have demonstrated successful clinical treatment through targeted B cell therapies in RRMS 1, 4-6.

## B cell dependent mechanisms in Multiple Sclerosis

Although MS has been considered as an autoreactive T cell mediated disease for a long period of time, accumulating evidence support the implication of B cells and antibodies in MS pathogenesis 7, 8. The oligoclonal bands that are detected after analyzing the cerebrospinal fluid (CSF) of MS patients, the presence of B and plasma cells in MS plaques and the accumulated antibodies and complement components in MS lesions emphasize the important role of B cells in MS pathogenetic mechanisms 8.

As principal mediators of the pro-inflammatory response that leads to the evolution of MS lesions were considered to be promulgated by the CD4+ T cells which produce IFN-γ 9. However, there are also other pathogenic cell subsets which are under the control of Th17 cells, that produce proinflammatory IL-17, IL-6 and are regulated by IL-23 10, 11. These can effectively recruit more T (CD4+, CD8+, γδ+), B, dendritic, natural killer (NK) cells and activate microglia in the perivascular space 9. Additionally, further evidence has revealed a loss of function and a decrease of induced FOXP3 expressing CD4+ CD25+ regulatory T cells (iTregs) subsets in patients with MS indicating their reduced suppressive potential towards the pathological inflammatory responses taking place 9, 12.

It is well established that the clinical biomarker detected in up to approximately 80% of patients has been found to have a CSF enrichment of with fragments of immunoglobulin G1 supporting the involvement of B cells in MS pathogenesis. Apart from the production of immunoglobulins, B cells can contribute to the pathology through cell interactions, their role as antigen presenting cells (APCs), that also present CNS antigens, proinflammatory cytokines (IFN-γ, TNFα, IL-6) producers 9, 13 or immune response modulators, that can restrict T cell functions through IL-10 directly or indirectly 14.

In patients with MS, B lymphoid follicles, T cells and APCs were identified in the meninges and complement was detected in MS lesions, underlying once again their role in the disease pathogenesis and particularly in the progression 9, 15. Concerning regulatory B cells (Bregs), their suppressive functions toward inflammation and immune responses by cytokine (IL-10, TGF-β, IL-35) secretion have not been clarified yet in particular in MS as they have been reported to be decreased, increased or even stable 13. However, elevated concentrations of the new CD19+ CD25+ type of Breg cells were observed in MS cases in direct contradiction to healthy controls and in relapsing phases compared to MS remissions 13.

It has been shown that the levels of cortical demyelination, meningeal inflammation and the consequent disability are all in direct correlation to B cell follicle-like structures (FLS) in the meninges of patients with progressive MS 14 that have been found near parenchymal infiltrates and underlying grey matter lesions 16. A study of patients with SPMS revealed the distribution of FLS in the forebrain, in the deep sulci of the frontal, temporal, insula and cingulate cortex as well as demyelinated lesions of subpial grey matter at some distance away from these foci 17. According to the “germinal center reaction” hypothesis naïve B cells recruited in the CNS (CXCL13 signaling) may present CNS antigens to Th cells. B and T cells then participate (as centroblast and follicular Th, that produce IL-21) in the formation of germinal centers where B to plasma cell maturation takes place and survival factors, that include B cell-activating factor (BAFF) are actively produced 14. FLS exist either as tertiary/ectopic lymphoid follicles in the case of chronically inflamed organs in autoimmune diseases such as MS, systemic lupus erythematosus (SLE) and rheumatoid arthritis (RA) or as embryological secondary follicles. In the FLS microenvironment of the brain, B cell interactions can influence/sustain humoral and cell immunity intensifying the already existent demyelination, oligodendrocyte loss and brain cortex damage 15 with the help of many activated microglial cells in the outer cortical layers. The inflammatory microenvironment is accompanied by neuronal loss and glial cell damage, mainly in the superficial cortical lamina, close to the outer pial surface 17.

A very important molecule, that acts as a regulator of B cells survival and development, is BAFF (or BlyS), a cytokine that belongs to the TNF family. It is produced by different cell populations, that can include macrophages, monocytes, dendritic, other immune cells and of course B as well as T cells 8, 18, 19. BAFF mediates its functions mainly through direct ligand-specific effects of three different receptors, thereby activating various signaling pathways, but it can also be involved in the development of autoimmunity 20.

Evidence for B cell involvement during neuroinflammation has also emerged from mice following EAE induction. This animal model represents the major hallmarks of the MS disease and pathology primarily through a CD4+ T cell-mediated autoimmune reaction that follows the MBP, PLP or MOG immunization 9, 21, 22 although CD8+ T cells constitute the predominant T cell populations in MS 23. This is a limitation of the current animal models that are utilized to understand the complexity of MS pathology since CD8+ T cells (touted as tissue resident memory cells) are observed to be compartmentalized within demyelinating lesions as CD20+ B cells are and CD4+ T cells seem to be transient through the course of disease 24. Indeed, if targeted CD20+ B cells therapeutics are administered in patients with RRMS we can achieve a reduction in CD8+ T cells that are myelin specific 25. Therefore, there is a need to model the complex immunopathogenesis of MS demyelination. Models that may resemble these patterns of immune cell-associated lesion profiles can be observed in the Theiler's Murine Encephalomyelitis Virus (TMEV) where the chronic progressive disease course is associated with potentiated myelin reactive CD4+ and CD8+ T cells 26 or the adoptive transfer of CD8+ autoreactive T cells from humanized mice that target PLP to produce profound brain inflammation 27. The unique interactions however between the innate and compartmentalized adaptive immune cells have not been clearly elucidated and are required to identify highly effective therapeutics that can modulate the demyelinating lesion milieu.

Even though research has been primarily focused on T cell-dependent pathogenesis lately, it is equally important to study the B cell mediated humoral responses in order to better understand the orchestrated immune responses taking place in conditions such as MS. Most versions of EAE lack the involvement of lymphocytes such as B cells in the disease. Nevertheless, administering larger protein antigens or even using B cells in non-immunization-based models can overcome such limitations 28. In this context, a study was conducted to reveal how B cells can participate in spontaneous CNS autoimmune disease of 2D2 IgH MOG mice, where spontaneous EAE can be a monophasic or chronic disease, correlated with T and B cell clusters formed within meninges and thereby inflammation in the spinal cord 28. Other than that, it was shown that in the EAE animal model without CX3CR1, a receptor for the NK cell recruitment with in the CNS, had elevated levels of CD4+ T cells and thus a more severe form of EAE. These findings suggest a possible ameliorating function of CX3CR1 and NK cells in the disease, regulating the levels of CD4+ T and Th17 cells 9.

## The regulation of BAFF during MS and other autoimmune diseases

High concentrations of BAFF have been observed in patients with various autoimmune diseases, such as RA, SLE, Sjogren's syndrome, systemic sclerosis, as well as in patients with myelin oligodendrocyte glycoprotein antibody (MOG-Ab) associated demyelination (a separate disease from MS and aquaporin-4 IgG-positive NMO) 8, 29. In the later case, patients had increased levels of B cell related cytokines such as BAFF that were associated with elevated Th-17, neutrophil, humoral and lymphoid cytokines (CXCL13, 12, 19), indicating BAFF's role not only in B cell immunity but in other inflammatory mechanisms as well 29. Although the role of BAFF in MS has not been completely elucidated, its increased levels in CSF during relapses and expression in active MS lesions 30 confirm its pathogenic role in the disease. Moreover, in the humoral response against EBV and Mycobacterium avium (MAP) infection, BAFF levels were found to be inversely associated 29.

Chronic autoimmune diseases, such as SLE, Hashimoto thyroiditis, RA, MS are characterized by inflammatory sites with lymphoid neogenesis, which constitute pathological lesions 31, 32. These regions seem to be enriched with BAFF expression 18, 31. Additionally, BAFF is also increased, up to lymphatic tissue levels, in MS plaques and even more in astrocytes of active MS lesions 30. Furthermore, high amounts of BAFF protein and mRNA levels can be found in monocytes and increased mRNA levels in T and B cells of MS patients 8, 29, 31. Detecting an association between sCD163 (marker of activated monocyte/ macrophages) and BAFF levels in CSF supports the consensus that infiltrating macrophages (in meninges, choroid plexus or CSF) and activated microglia with in the brain are probably related to constant intrathecal production of BAFF and inflammatory mediators 29. It is considered that the infiltrating macrophages, microglia and dendritic cells might also be possible sources of BAFF, responsible for the proliferation of CNS invasive autoreactive B cells during inflammation 29.

From a genetic point of view, a correlation has been found between an insertion-deletion variant, derived from TNFSF13B, which encodes BAFF, and the higher risk for MS, SLE and possibly RA 33. The product of this mutation is a short transcript, the function of which is not inhibited resulting in excessive production of BAFF. The increased BAFF concentration is related to a rise in the population of B cells and antibodies, as well as to lower monocyte numbers and therefore a higher propensity exists for the development of autoimmunity 33.

In mice with relapsing-remitting and chronic relapsing EAE the CNS was characterized by a higher expression of BAFF and CXCL13 genes 7. Follicular dendritic cells, in secondary lymphoid organs, produce CXCL13 to lead B cells into their follicles 34-36. It has been found that, during EAE, CXCL13 and BAFF mRNAs are induced intracerebrally and CXCL13-producing cells can be detected in the meninges of these mice suggesting that these tissue sites may be responsible for inducing autoimmune disease 7.

Another study in BAFF-deficient mice revealed that treating the animals with a recombinant Fc-BAFF protein allows splenic B cell zone formation, B cell maturation and physiological antibody response against T-dependent antigen to occur. These functions were limited whenever the treatment was not applied and the mature B cell population was estimated to be significantly lower. Thus, administering recombinant Fc-BAFF can be used to treat BAFF-deficiency 37.

## BAFF system: structure, function, signaling pathways and the association with NgR and autoimmunity

The two putative ligands, BAFF and A proliferation inducting ligand (APRIL), as well as their three cognate receptors (BAFF-R, TACI and BCMA) cooperatively regulate the BAFF cell signaling pathways. BAFF, also known as BlyS, THANK-1, CD257, TNFSF13B, ZTNF4 and TALL-1, was first detected in 1999 by multiple research groups 38-40. It is a type II transmembrane protein that consists of 285 amino acids and belongs to the tumor necrosis factor (TNF) family of ligands 38, 41. The protein is encoded in human by the BAFF gene on chromosome 13 (13q34) 38, 42, 43 with exons: 1, 2 and 3-6 coding for the transmembrane domain and its flanking regions, the furin processing site and the TNF homology domain (which binds to receptors), respectively 38. As a member of the TNF family BAFF's main functions are related to cell death, differentiation and survival 8. The molecule is of great importance for B cell survival, development, differentiation, immunoglobulin production and T cell stimulation 29.

BAFF is found initially as a homotrimer on the cell membrane, but if it gets cleaved by furin protease, it is released as a soluble trimeric cytokine 19, 44. Even though its receptor-binding domain is trimeric, it is not uncommon for 20 BAFF trimers to assemble through a loop region (flap) into a 60-mer 45, 46. There is also delta-BAFF, a short variant protein, which instead of connecting with BAFF-R, binds to BAFF resulting in inactive heterotrimeric products and therefore negative regulation of BAFF can ensue 47. Apart from the homotrimers and delta-BAFF, BAFF can form polymers of various compositions with its structural homolog 8, APRIL 38, which are susceptible to BAFF-targeting therapies 46. APRIL (also known as TRDL-2, TNFSF-13A, TALL-2 38) exists mainly as a soluble molecule 8, but it can be localized to plasma membrane surfaces as part of the hybrid molecule TWE-PRIL (also known as TNFSF12-TNFSF13), derived from TWEAK (TNF-related weak inducer of apoptosis) and APRIL gene trans-splicing 47, 48.

BAFF production has been observed in most myeloid cells and innate immune cells like macrophages, neutrophils, monocytes, IL2‐activated natural killer cells and TLR9‐activated plasmacytoid dendritic cells 18, B and T cells 44, 49 and radiation-resistant stromal cells 18, 48. APRIL is mainly produced by neutrophils, macrophages, monocytes, dendritic cells and T-cells 8. Additional BAFF and/or APRIL sources seem to be nonhematopoietic cells, like epithelial cells and astrocytes with a possible involvement in B cell maintenance at disease specific sites 18. Furthermore, BAFF can be detected in non-lymphoid cells such as salivary ductal cells in Sjogren's which might be a key pathogenic role in autoimmune epithelitis 50, airway gland epithelial cells where BAFF and APRIL expression may contribute to local migration, class switch recombination and Ig production by B cells found in the airway 51 or osteoclasts (also express APRIL) 52. Complementary, vascular cell adhesion molecule 1-positive stromal bone marrow cells (VCAM-1 BMCs) that express the molecule seem to be a cell population that enhances developing B and plasma cells in bone marrow 53 and fibroblast-like synoviocytes that are induced by cytokines like IFN-γ and TNF-α to express BAFF may be highly capable to protect B cells from apoptosis in inflammatory microenvironments *in vivo* 52, 54.

The main cognate cell-surface receptors are BAFF receptor-(BAFF-R), the transmembrane activator, calcium-modulator and cyclophilin ligand-(TACI) and the B-cell maturation antigen-(BCMA) 8, 55, 56. In humans, all three of them are found on B cells. BAFF-R is involved in B cell survival, maturation and tolerance 20, 42, 47, 57 being expressed on all B cells apart from bone marrow plasma cells, as well as on Tregs and activated T cells 47, 52. TACI is important for T cell-independent responses of B cells to type I and type II antigens, negative regulation of the B cell class-switch recombination and arrangement 46. This receptor can be detected on tonsillar and bone marrow plasma cells, activated CD27- non-germinal center cells, CD27+ memory B cells and naive B cells in tonsils and blood 47. Human intracellular TACI is expressed by macrophages and after being activated it is found on the cell surface 52. Evidence exists in support of TACI's capability of inducing B cell apoptosis and thus potentially having an important role in preserving immune system homeostasis 58, 59.

BCMA is produced by spleen, bone marrow 47, plasmablasts, plasma cells, tonsillar memory B cells and germinal center B cells 52 promoting survival 46. It has a significant role, especially in the survival of bone marrow plasma cells and plasmablasts, at later stages 60, 61 and its complex with BCMA can overpower BAFF in the last stages of differentiation 42. APRIL, on the other hand, interacts with TACI, BCMA and surface heparin sulfate proteoglycan (HSPG) leading to its multimerization and subsequent downstream signaling 44.

BAFF and BAFF-R pathways target canonical or non-canonical NF-κB dependent pathways. The canonical NEMO-dependent pathway requires the IKK complex, activators such as TRAF2, 5 and 6 and yields NF-kB dimers with a p50 subunit 42, 57, 62, 63. Stimulation of the non-canonical NF-κB2 pathway by BAFF is slow, complex 64 and it depends on the NF-κB inducing kinase NIK (MAP3K14) activation 20, 65. If BAFF is not present, NIK binds TRAF3, promoting the proteasomal degradation of NIK by a complex of the cellular inhibitors of apoptosis cIAP1 and 2, TRAF2 and 3. Conversely, when BAFF binds to BAFF-R, the receptors cluster and TRAF3 binds to their intracellular sequence allowing the NIK-TRAF2/3-cIAP1/2 complex disassembly and the TRAF3 proteasomal breakdown 20. The accumulated NIK molecules phosphorylate IKK1, which phosphorylates NF-κB2 p100, the new target of a ubiquitin ligase, that is cleaved into active p52. P52 binds with relB and can regulate nuclear genes, like ICOSL, a costimulatory ligand for ICOS, from activated T cells, that promotes follicular Th cells development 20.

**Figure 1:**
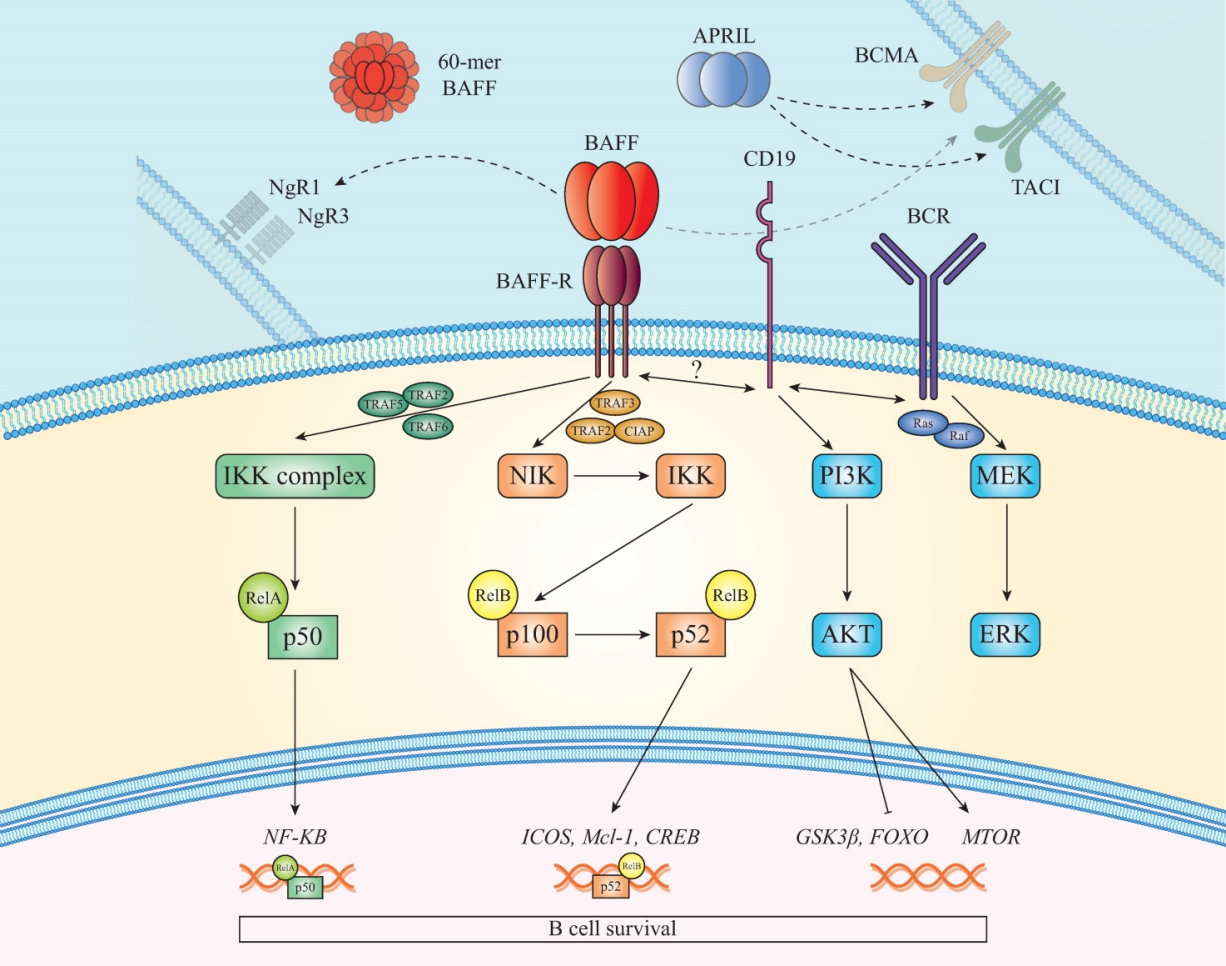
** BAFF and BAFF-R signaling pathways.** In the presence of BAFF, TRAF3 is intracellularly recruited to BAFF-R allowing NIK to activate the non-canonical NF-KB2 pathway which results in p52/RelB nuclear translocation and activation of cell survival genes. Another pathway activated after BAFF/BAFF-R binding is the canonical that upregulates the expression of various genes, such as NF-KB. Apart from these, PI3K pathway, which shares molecules with the BCR signaling, is additionally activated. Furthermore, it has been shown recently that BAFF interacts with NgR1 and NgR3, promoting B cell proliferation and maturation. Lastly, TACI and BCMA can also bind with BAFF and APRIL firing signals that promote antigen presentation and cell survival.

Another pathway associated with BAFF-R is the phosphoinositide-3-kinase (PI3K) dependent signaling cascade, which shares common components with the BCR pathway 66, 67. BAFF-R signals can interplay with various molecules (WIP, CD81 and CD19), resulting in cytoskeleton modification. Downstream of this pathway, the AKT/mTOR axis activates B cell-specific metabolic reactions involved in their survival and function. In mice, BAFF triggers phosphorylation of AKT 68 and subsequently of the factor FOXO, the inhibitor 4EBP1, GSK3β and the small ribosomal subunit protein S6 indicating BAFF-R significance in protein synthesis. Moreover, MCL-1 stabilization 68, leads to increased mitochondrial function and ATP production 20 (see **Figure 1**).

BAFF signaling is also involved in the recombination for immunoglobulin class switching. Even though in the case of T-dependent antigens it requires at least two independent signals, from cytokines and also CD40 42, 69, in human B cells there is no need for additional CD40 signals with BAFF and APRIL being the main ligands 42, 70. There is, also, evidence about the B cell co-receptor complex (CD19, CD21 and CD81), which may be enhanced by BAFF, connecting to C3 complement component. Opsonized antigens are able to cross-link the BCR to the co-receptor, reducing the point of B cell activation and accelerating T-independent antibody production 42. BAFF and its cognate receptor are also implicated in T cell physiology inducing the proliferation of suboptimally stimulated T cells 42. BCMA, which primary B cells can obtain under collaborative IL-4 and -6 effects, is associated with increased antigen presentation 42. Regarding TACI, its presence is important for communication to occur between B cells and BAFF-expressing dendritic cells which will eventually allow dendritic cells to prime CD8+ T cells 42, 71.

Changes in circulating levels of BAFF, which are genetically regulated and lead to its increase, seem to be strongly correlated with the risk of autoimmunity, as Genome-wide genetic association (GWAS) studies on MS and SLE patients reveal 20. Moreover, restricted or limited TACI function can also lead to immunodeficiency or autoimmunity accompanied by lymphoproliferation, thereby TACI deficient humans and mice can develop since BAFF levels no longer are properly regulated 20. Many animal models representing diseases do exhibit high levels of soluble BAFF. In the case of chemically induced autoimmunity by HgCl_2_ there is an elevated production of BAFF, and TACI-Ig fusion protein is a suggested therapeutic that neutralizes both BAFF and APRIL inhibiting Hg-induced autoantibody or IgE secretion 72. In collagen-induced arthritis the overproduction of BAFF by dendritic cells and macrophages may play a crucial role in the pathogenesis of the experimental arthritis 73. Lastly, in SLE models, BAFF and its receptors are involved in the development of the disease and TACI-Ig seems to be a promising treatment, potentially applicable in human autoimmune disease 74. About (20-50%) of individuals living with autoimmune diseases have been reported to also exhibit elevated levels of BAFF which have been correlated with pathogenic autoantibodies and disease severity. Indeed elevated APRIL and BAFF/APRIL heterotrimers has been observed in sera, targeting patients' organs and thus indicating a strong possibility for excessive BAFF and APRIL to have a pathogenic role in autoimmune sequelae 52.

## NgR complex, signaling pathways, interaction with various components

It has previously been revealed that there is a correlation between BAFF and Nogo receptor (NgR) signaling complex 75, a member of the leucine rich repeat (LRR) superfamily 76, that binds with high affinity and may potentially function as a BAFF receptor 48, 77. The NgR signaling complex consists of NgR1, the co-receptor LINGO-1 and the neurotrophin receptor p75 (p75NTR) or TROY receptor and it mediates its inhibitory effect against axonal regeneration through binding to Nogo 66 with nanomolar affinity 78-80. This receptor complex binds with myelin associated inhibitory factors (MAIFs), which are myelin-associated glycoprotein (MAG), oligodendrocyte-myelin glycoprotein (OMgp) and Nogo-A and are found on myelin sheath of oligodendrocytes 77, 81, 82. Nogo-A is one of the three splice variants (Nogo A, B and C), that have a conserved extracellular region of 66 amino acids 80, 83, which is found between two transmembrane helices allowing the molecule to anchor to the oligodendrocyte membrane 81. This region is responsible for the Nogo related signals that inhibit neurite outgrowth and promote growth cone collapse 79, 81, 84.

Apart from neurons, NgR is also detected on immune cells. More specifically, NgR1 can reduce synaptic plasticity in the cortical gray matter and modulate dendritic spines that are experience dependent 85. LINGO-1, another LRR superfamily member, is found in CNS neurons and oligodendrocytes inhibiting oligodendrocyte differentiation and myelination 71, 78. The low affinity neurotrophin receptor p75NTR, is expressed in a plethora of cell types 78. Its functions comprise cell proliferation, apoptosis, and interestingly the NgR1 and LINGO-1 synergistically inhibit axonal regeneration. Another receptor expressed in neurons is TROY, which substitutes p75NTR within the NgR complex under certain conditions and is also involved in axon growth suppression 78.

Following the high affinity ligation of the MAIF proteins with NgR, alpha and gamma secretase activity can physiologically modify signaling through intramembranous cleavage of p75NTR (or alternatively TROY 86) liberating an intracellular signaling peptide, that suppresses axonal growth through activation of Rho GTPase and hence its Rho kinase to limit microfilament and microtubule polymerization 81. Additionally, gangliosides play a collaborative role in the signaling complex and have also been demonstrated to be important in preventing neuronal growth after spinal growth injury 80, 81. During neuroinflammation it has been demonstrated that NgR1-dependent signaling within spinal cord and optic nerve axons occurs through RhoA-GTP and the phosphorylation of the Rho-associated coiled-coil containing protein kinase 2 (ROCK II) to alter axonal transport and elicit neurodegeneration through downstream phosphorylation of the collapsin response mediator protein 2 (CRMP2) at the threonine 555 site located at its C-terminus 21, 87. Moreover, activation of NgR signaling through the binding of MAIFs present in extracellular myelin debris, can potentiate endogenous inhibitory effects on the maturation of oligodendroglial precursor cells (OPCs) and their recruitment around demyelinated plaques disrupting the remyelination process in progressive MS 77.

Recently, a study revealed that NgR expression in the CNS of EAE mice may be involved in BAFF signaling pathways which promote B cell proliferation, maturation and differentiation in EAE lesions 77. The evidence suggests that NgR1+ and NgR3+ B cells, mainly found clustered in leptomeningeal infiltrates of lumbosacral spinal cords, can produce antibodies which target spinal cord white matter myelin and the integral myelin membrane glycoproteins 77. Even though BAFF and NgR1 binding can result in DRG neurite outgrowth inhibition, it may also lead to the expansion of a subclass of intrathecal NgR1+ and NgR3+ B cells which can propagate neuroinflammatory damage. Thus, it was shown for the first time, that NgR1 and NgR3 contribute actively in B cell maturation during EAE and may be involved in BAFF and various other signal transduction events that lead to intrathecal proliferation of these cells. A finding that is also supported by inhibition of B cell proliferation and differentiation that can result after blocked BAFF binding to NgR as it was shown that it can be antagonized by the Nogo receptor fusion proteins (NgR-Fc and NgR3-P) 77. These findings are in agreement with the possible indication of BAFF's involvement in the pathophysiological mechanisms of various autoimmune CNS diseases as increased expression was identified in astrocytes and microglia in patients with MS 38, 75.

Moreover, chondroitin sulphate proteoglycans (CSPGs) from reactive astrocytes or T-cells can promote B-cell differentiation and involve in antigen presentation during autoimmunity or even modulate B-cell maturation through binding and activating APRIL. This is in agreement with the fact that NgR1 and NgR3 can interact with many receptors inducing various signaling pathways for B-cell activation, which is of great significance in MS since its histopathological hallmark indicates the presence of autoreactive B-cells in follicles within the meninges and can lead to progressive neurodegeneration 77. Furthermore, BAFF seems to be a possible candidate for therapy in MS, since it is produced by astrocytes of the CNS during its pathogenesis and it is associated with cells that have BAFF-R on their surface which is up-regulated in meninges of ectopic lymphoid follicles. Thus, antagonizing BAFF and the following signaling cascade with recombinant NgR peptides may be one way to inhibit the B-cell proliferative response 77.

## Can the therapeutic targeting of BAFF and NgR be developed to limit B cell proliferation/maturation and neurodegeneration in MS?

The development of the modern MS treatments begins with IFNβ and glatiramer acetate which were used for the reduction of relapses in relapsing - remitting MS (RRMS) cases 88-91, of white matter lesions development and disability accumulation 92, 93. Monoclonal antibodies such as natalizumab that inhibits lymphocyte invasion in the CNS after blocking adhesion through α4β1 integrin, lately used oral medications fingolimod, ozanimod and siponimod that are sphingosine-1-phosphate (S1P) receptor regulators capable of separating lymphocytes in primary lymphoid organs and teriflunomide, anti-inflammatory dimethyl fumarate, cladribine, mitoxantrone are only some of the recent additions 88-92, 94. The development of new monoclonal antibodies, like alemtuzumab, as well as B cell depleting therapies with anti-CD20 monoclonal antibody rituximab and ocrelizumab, ofatumumab, anti-CD19 antibody inebilizumab 95 continues enriching the list of currently approved therapies 88-91, 94, 96 (**Table 1**).

In this context, could the therapeutic targeting of BAFF limit the damaging effect of proliferating B cells in MS? This question seems to concern scientists/clinicians and it has become a focus of emerging research pathways in B cell depleting MS therapy, since BAFF as well as APRIL potentiate B and plasma cells survival. Experiments with BAFF antagonists have demonstrated favorable outcomes in murine models of RA, Graves' disease and MS, enhancing their appeal as possible treatments for human patients 52, 97. Despite these positive experimental investigations and the clinical data which show that anti-CD20 monoclonal therapeutic antibodies deplete B cells, targeting BAFF and APRIL has important effects on mature plasma cells, since these populations express BCMA but not CD20. Furthermore, rituximab (a well utilized therapeutic agent to reduce CD19+ B cells) can elevate serum BAFF levels, increasing the possibility of immature cells being exposed to high levels of BAFF and leading to autoimmunity. These side effects could be averted by targeting BAFF directly 52.

For this purpose, depleting anti-BAFF monoclonal antibodies and soluble TACI-Fc fusion protein atacicept, which diminish BAFF and APRIL, have been investigated 98. Early phase clinical trials revealed that atacicept worsened the disease course without a clear etiology. The increased activity in patients indicated that the protective role of B cells was affected underlying the complexity and the difficulty to target humoral immunity in MS 97-100. On the other hand, belimumab, a BAFF antibody, has been used in SLE patients with B cell occurring seropositivity 97, 101 and clinical trials have been conducted with tabalumab/ LY2127399 antibody for RRMS, although no indication of treatment effect was confirmed 97, 98. In the EAE model, a BAFF - APRIL antagonist (soluble B-cell maturation antigen-Fc) improved the disease course as it was expected 97, whereas mice without BAFF-R had enhanced damage 98.

Although the current MS treatment field seems to be dominated by immunomodulatory therapies, cases of chronic active MS lesions are unresponsive to them underlying an imperative need for new agents, able to modify neurodegenerative lesions 77, 102. It is worth noting that our recent data derived from the EAE model may partly elucidate the effect of the BAFF protein as a ligand for Nogo receptor (NgR1 and NgR3) expressing B cells. These cells can proliferate and differentiate under the control of BAFF, although provision of the NgR fusion protein can antagonize this effect 77. Moreover, such cells, located within leptomeninges, are capable of secreting anti-myelin antibodies whose targets are myelin and oligodendrocytes 77. The data support that BAFF signaling pathways are involved in neuroinflammation and both NgR1 and NgR3 can be of great significance for the neurodegenerative mechanisms that govern the progressive outcomes observed in EAE and MS, suggesting that specifically targeting this interaction on maturing B cells could be a possible target for novel treatment approaches in MS 77.

Collectively, B cell therapy is still in its infancy and the complexities of pathogenic signaling need to be resolved to initially understand these proinflammatory subpopulations, maintaining the protective - regulatory cells 95 and even how we can target the neurodegenerative changes. BAFF binding to NgR may indeed be a plausible target for chronic active MS treatment. Since the BAFF and APRIL system can interact with components of T and B cell pathways of chronic inflammatory disorders targeting a novel signaling cascade that may potentiate autoimmunity warrants further investigation. Considering that NgR1+ and NgR3+ B cells maybe involved in humoral mediated neuroinflammatory events, it is clear that therapeutic treatments against the immune responses that are myelin orientated may be developed.

## Figures and Tables

**Table 1:** Approved immunomodulatory therapies for MS

Brand name	Generic name	Target	Function
Betaferon (Bayer)	Interferon-β-1b	Interferon-a/b receptor	Inflammatory regulation
Copaxon (CSL)	Glatimerar acitate	MBP reactive lymphocytes	Prevent myelin specific responses by lymphocytes
Rebif (Merck)	Interferon-β-1a	Interferon-a/b receptor	Inflammatory regulation
Avonex (Biogen)	Interferon-β-1a	Interferon-a/b receptor	Inflammatory regulation
Tysabri (Biogen)	Natalizumab	a4-integrin	Reduces lymphocyte trafficking through BBB
Gilenya (Novartis)	Fingolimod	Sphingosine-1 phosphatase receptor	Limits lymphocyte trafficking out of lymph nodes
Tecfidera (Biogen)	Dimethyl fumarate	Unknown	Protective against oxidative stress
Aubagio (Genzyme)	Teriflunomide	Dihydroorotate dehydrogenase	Limits immune cell replication
Lemtrada (Genzyme)	Alemtuzumab	CD52+	T and B lymphocyte depletion
Plegridy (Biogen)	Pegylated Interferon-β-1a	Interferon-a/b receptor	Inflammatory regulation
Ocrevus (Roche)	Ocrelizumab	CD20+ lymphocytes	Inflammatory regulation reducing CNS attacks
Mavenclad (Merck)	Cladribine tablets	DNA of proliferating immune cells	inflammatory regulation
Mayzent (Novartis)	Siponimod	Selective binding of sphingosine-1-phosphatase receptor	Prevents immune cell egress from lymph node
